# Bias in Care: Impact of Ethnicity on Time to Emergent Surgery Varies Between Subspecialties

**DOI:** 10.5435/JAAOSGlobal-D-23-00060

**Published:** 2023-06-13

**Authors:** Sarah R. Blumenthal, George W. Fryhofer, Viviana Serra-Lopez, Sarah N. Pierrie, Samir Mehta

**Affiliations:** From the University of Pennsylvania, Philadelphia, PA (Dr. Blumenthal, Dr. Fryhofer, Dr. Serra-Lopez, and Dr. Mehta) and Brooke Army Medical Center (Dr. Pierrie), Fort Sam Houston, TX.

## Abstract

Disparity in access to emergency care among minority groups continues to exist despite growing awareness of the effect of implicit bias on public health. In this study, we evaluated ethnicity-based differences in time between admission and surgery for patients undergoing emergent procedures at hospitals participating in the American College of Surgeons National Surgical Quality Improvement Program.

**Methods:**

We conducted a retrospective review of 249,296 National Surgical Quality Improvement Program cases from 2006 to 2018 involving general, orthopaedic, and vascular surgeries. Analysis of variance was used to compare “time to operating room” (OR) between ethnic groups.

**Results:**

Notable differences in time to OR were noted among general and vascular surgeries but not orthopaedic surgery. Post hoc comparison identified notable variation in general surgery between White and Black/African Americans. In vascular surgery, notable variations were identified between White and Black/African Americans and White and Native Hawaiian/Pacific Islanders.

**Discussion:**

These findings suggest that certain surgical subspecialties continue to exhibit disparities in care that may manifest as surgical delay, most notably between White and Black/African Americans. Interestingly, variation in time to OR for patients treated by orthopaedic surgery was not notable. Overall, these results highlight the need for additional research into the role of implicit bias in emergent surgical care in the United States.

Disparity in access to emergency medical care among minority groups in the United States continues to exist despite a growing awareness of the effect of implicit bias on public health. Even with multiple national initiatives throughout the years aimed at reducing disparities and achieving health equity, progress has been slow.^1,2^ Black patients remain markedly more likely to die in the hospital than White patients after surgical procedures.^3^ These gaps manifest across specialties. For instance, mortality among Black patients who undergo anterior cervical spine surgery is 1.57 times higher than for White patients, even after adjusting for other demographic characteristics, including age, insurance, and geographic region.^4^ Similarly, in-hospital mortality after coronary artery bypass grafting in 2014 measured 4.2% among Black Medicare patients compared with 3.3% among White patients.^5^

The type of surgical care provided to minorities also varies from that provided to White patients. Medicare data have demonstrated that Black patients are much less likely to have undergone attempts at limb revascularization before amputation than White patients.^6,7^ Outpatient clinical settings are vulnerable to these same issues. Patients who are Hispanic and non-Hispanic Blacks, along with those of lesser education status, with lower income, or with public insurance, exhibit lower rates of utilization of outpatient office-based orthopaedic care for musculoskeletal conditions, with greater reliance on the emergency department.^8^ Black and “non-White” patients with peripheral vascular disease and hyperlipidemia are more likely to receive primary care at community health centers where they are markedly less likely to receive cardiology consultations.^9,10^

Emergency settings are particularly susceptible to implicit biases and socioeconomic discrepancies.^11^ Black and Hispanic patients are more likely to be transported to a safety-net designated emergency department in major US cities than their White counterparts.^12^ Once in an emergency department setting, Black patients are less likely than White patients to receive analgesia for acute pain.^13^

Defining the barriers associated with these discrepancies remains elusive. Most existing literature focuses on outcomes data while overlooking antecedent factors such as prehospital care and delays in treatment. In this study, we evaluated whether ethnicity-based differences exist in time between admission and surgery for patients undergoing emergent procedures at hospitals participating in the American College of Surgeons National Surgical Quality Improvement Program (NSQIP).

## Methods

We conducted a retrospective review of all available American College of Surgeons NSQIP data from 2007 to 2018. We selected the three surgical specialties with the highest number of overall cases, with specialty defined by the principal surgical procedure for a given case. The three highest volume surgical specialties were general surgery, orthopaedic surgery, and vascular surgery. Cases were evaluated both in aggregate and by Current Procedural Terminology (CPT) codes, which were used to identify the top 10 most commonly conducted procedures among each of these specialties. Only cases designated by NSQIP as “emergent” were included. This designation is applied to cases in which “the patient's well-being and outcome is potentially threatened by unnecessary delay and the patient's status could deteriorate unpredictably or rapidly.”^14^

Ethnic groups identified as distinct populations by NSQIP were American Indian/Alaskan Native, Asian, Black/African American, Native Hawaiian/Pacific Islander, and White. Cases where the patient race was “unknown” or “not reported” were excluded from analysis. A separate analysis was conducted on patients designated as Hispanic or non-Hispanic because the NSQIP database uses this description as a separate variable. Cases in which Hispanic ethnicity was not designated were assumed to be non-Hispanic.

The primary outcome for this study was mean time from patient's hospital arrival to the start of the procedure in the operating room (OR), measured in days.

## Statistical Analysis

Analysis of variance was used to compare “time to operating room” (OR) among the different ethnic/racial groups for all emergent cases within each specialty, as well as for the 10 most frequent CPT codes for each specialty. Two-tailed Student t-tests were used for comparisons of non-Hispanic versus Hispanic patients.

Post hoc comparison of time to OR relative to White ethnicity was done for procedural codes where the analysis of variance was notable. *P* value less than 0.005 was considered significant, based on Bonferroni correction for 10 comparison groups. The Strengthening the Reporting of Observational Studies in Epidemiology (STROBE) guidelines were used for reporting observational cohort studies.^15^

## Results

Among NSQIP data from 2007 to 2018, the three highest volume surgical specialties in case numbers were general surgery, orthopaedic surgery, and vascular surgery. Within these three specialties, 249,340 cases were identified as emergent procedures and were included in analysis. Demographic differences between White, Black or African American, Asian, American Indian or Alaska Native, and Native Hawaiian or Pacific Islander are provided in Table 1. Although multiple variables reached statistical significance between the groups, additional scrutiny revealed little clinical variability (eg, range of variability for each mean value listed between groups is minimal).

**Table 1:** By Ethnicity, Demographic Characteristics of Patients Undergoing Emergent Surgery, Including the Top 10 Current Procedural Terminology Codes for General, Orthopaedic, and Vascular Surgeries

	White	Black or African American	Asian	American Indian or Alaska Native	Native Hawaiian or Pacific Islander	*P*
	%, or mean (SD)	%, or mean (SD)	%, or mean (SD)	%, or mean (SD)	%, or mean (SD)	
Age (yrs)	51.2 (21.4)	48.5 (19.8)	48 (20.1)	43.4 (19.1)	43.7 (18.1)	<0.001
BMI (kg/m^2^)	28.3 (7)	29.7 (8.2)	25.1 (5.1)	29.9 (7.7)	29.9 (7.5)	<0.001
Sex						
Female	53.2	54.6	52.4	55.4	53.6	0.002
Male	46.8	45.4	47.6	44.6	46.4	
Functional status						
Independent	93.1	91.1	95.7	93.7	94.4	<0.001
Partially dependent	4.5	5.3	3	4.2	2.3	
Totally dependent	1.6	2.7	0.7	1	0.9	
Unknown	0.8	0.9	0.5	1.1	2.3	
ASA class						
1	19.8	15	32.6	15.2	23.4	<0.001
2	41.9	38.6	43.9	48.1	50.1	
3	26.4	30.4	17.5	26.6	19.6	
4	10.6	14.4	5.1	8.7	4.4	
5	1.2	1.5	0.8	1.2	1.3	
None	0.1	0.1	0.1	0.3	1.2	
Anesthesia						
General	96.9	98.6	94.7	98.6	98.6	<0.001
Spinal	2	0.6	4.6	0.8	0.8	
Regional	0.1	0.1	0.1	0.1	0.5	
MAC/IV sedation	0.7	0.4	0.4	0.4	0.1	
Other	0.3	0.2	0.1	0.1	0	
Diabetes						
Non-diabetic	90.2	85	89.2	87.1	87.2	<0.001
Non-insulin	5	7.1	7.1	6.1	6.6	
Insulin	4.2	7.2	3.3	6.1	5.5	
Oral	0.5	0.7	0.4	0.6	0.7	
Current smoker	19.3	24.4	10.3	34.9	19.3	<0.001
History of COPD	5.1	3.9	1	4.2	1.4	<0.001
History of ascites	1.1	1.7	0.7	1.8	1.2	<0.001
History of CHF	1.4	2.2	0.8	1.3	1.2	<0.001
Dialysis	1	4	1.2	1.5	2.9	<0.001
Disseminated cancer	1.6	2.1	1.2	0.8	1.4	<0.001

SD = standard deviation, BMI = body mass index, ASA = American Society of Anesthesiologists classification, MAC = Monitored Anesthesia Care, and COPD = Chronic Obstructive Pulmonary Disease

Significant differences in time to OR among races were identified among emergent procedures in aggregate for general surgery (*P* < 0.001) and vascular surgery (*P* < 0.001) but not orthopaedic surgery (*P* = 0.095) (Table 2). Post hoc comparison identified significant variation in general surgery time to OR between White and Black/African American (1.03 days [White] vs. 1.36 days [Black] to OR, *P* < 0.001) populations. In vascular surgery, significant variations were identified between White and Black/African American (1.35 days [White] vs. 2.32 days [Black] to OR, *P* < 0.001) as well as White and Native Hawaiian/Pacific Islander (1.35 days [White] vs. 3.13 days [Native Hawaiian/Pacific Islander] to OR, *P* < 0.001) groups. Significant differences in time to OR between non-Hispanic and Hispanic patients were also identified among emergent procedures in general surgery (1.08 days [non-Hispanic] vs. 0.93 days [Hispanic], *P* = 0.005) and vascular surgery (1.49 days [non-Hispanic] vs. 2.29 days [Hispanic] to OR, *P* < 0.001) but not orthopaedic surgery (*P* = 0.645) (Table 3).

**Table 2:** Analysis of Variance Results Comparing Mean Time to Operating Room Between White and Minority Populations for Top Three Surgical Specialties in the National Surgical Quality Improvement Program, Measured in Days

Specialty	N	White		Black or African American		Asian		American Indian or Alaska Native		Native Hawaiian or Pacific Islander		*P*
		Mean	(SD)	Mean	(SD)	Mean	(SD)	Mean	(SD)	Mean	(SD)	
***General surgery***	208125	**1.03**	6.88	**1.36** ^a^	5.76	0.93	5.57	0.78	2.81	0.93	2.92	**<0.001**
*Orthopaedic Surgery*	22649	1.03	5.06	1.44	10.11	0.92	2.46	0.84	1.34	0.97	1.85	0.095
***Vascular surgery***	18522	**1.36**	4.88	**2.32** ^a^	8.82	1.83	5.02	1.36	2.67	3.13*	10.38	**<0.001**

a Indicates significant difference (threshold *P* < 0.005) detected relative to the White group on post hoc testing. Bolded text denotes findings that meet threshold for statistical significance.

**Table 3:** Analysis of Variance Results Comparing Mean Time to operating room (OR) Between Non-Hispanic and Hispanic Populations for Top Three Surgical Specialties in the National Surgical Quality Improvement Program, Measured in Days

Specialty	N	Non-Hispanic		Hispanic		*P*
		Mean	(SD)	Mean	(SD)	
***General surgery***	208125	**1.08**	6.49	**0.93** ^a^	7.9	**0.005**
*Orthopaedic Surgery*	22649	1.05	5.56	1.08	1.71	0.645
***Vascular surgery***	18522	**1.49**	5.73	**2.29** ^a^	4.29	**<0.001**

a Indicates significant difference (threshold *P* < 0.005) detected relative to the White group on post hoc testing.

Subanalysis of the top 10 CPT codes within each specialty (Supplemental Table 1, http://links.lww.com/JG9/A286) demonstrated that general surgery procedures with significant ethnicity-based differences in time to OR were partial colectomy and surgical laparoscopy. In vascular surgery, notable differences occurred with endovascular repair of infrarenal abdominal aortic aneurysm, embolectomy/thrombectomy, thromboendarterectomy, and below-knee amputation. Of note, although ethnicity-based variation among all orthopaedic surgery procedures did not reach statistical significance, a significant difference was noted in CPT subanalysis for trimalleolar ankle fracture open reduction and internal fixation between White and Black/African American populations (0.48 days [White] vs. 2.28 days [Black] to OR, *P* < 0.001). Subanalysis did not demonstrate significant differences for individual CPT codes between non-Hispanic and Hispanic groups (Supplemental Table 2, http://links.lww.com/JG9/A286).

## Discussion

Many factors influence timing of emergent surgical procedures done at hospitals in the United States. Some of these preoperative considerations and patient characteristics lead to unavoidable delays while others may reflect implicit biases that shape disparities in patient care. Defining the risk factors that influence poor outcomes may affect future work to close unperceived gaps in patient care. While data consistently demonstrate that Black race predicts higher mortality after trauma independent of socioeconomic status, questions remain regarding the exact etiology of contributing factors.^16^

There is a paucity of literature to date focusing on prehospital care, yet disparities in access are undeniable. Minority patients, along with lower education or socioeconomic status, are markedly more likely to live in areas that lack access to emergency general surgery care.^17^ Although the clinical significance is unclear, frequency of interhospital transfer also varies based on race and insurance status.^18,19^

When evaluating elective surgeries only, there is conflicting research in the orthopaedic literature on the effect of ethnicity-based bias on patient care. In one study, the authors used computerized scenarios supplied to a group of surgeons recruited from the 2012 annual meetings of the New York State Society of Orthopaedic Surgeons and the American Association of Hip and Knee Surgeons. They found that race did not influence orthopaedic surgeon's recommendations for or against total knee arthroplasty and concluded that physician bias may be less influential in circumstances where strong clinical data are used to support decision making.^20^ Contrarily, prior large-database retrospective analyses have demonstrated higher rates of perioperative complications and discharge to non-home facilities in Black patients after total hip arthroplasty.^21,22^ Furthermore, a higher percentage of Black patients who undergo primary total hip arthroplasty return to the emergency department to receive unplanned hospital services for complications within 30 days.^23^ Altogether, these data suggest that there may be more complex factors contributing to a differential influence of ethnicity in various practice settings.

In this study, we demonstrate a notable difference in time to OR among patients of different ethnicities undergoing emergent general surgery and vascular surgery procedures, most notably between White and Black/African American populations. Interestingly, variation in time to surgical treatment of all patients treated by orthopaedic surgery was not notable, which may reflect the unique weight given to objective radiographic findings within this specialty and defined, evidence-based treatment algorithms (eg, urgent fixation with an intramedullary nail or sliding hip screw for geriatric intertrochanteric fractures, urgent intramedullary nailing for femoral shaft fractures). This data point is consistent with the above-referenced literature supporting a reduced influence of bias where strong clinical data endorse surgical treatment.

This study does not account for case specifics such as injury severity that fall outside the scope of the NSQIP data set but may also contribute to variation in time to OR. It is also limited in its ability to differentiate results for Hispanic and non-Hispanic patients because of the separate variable that does not account for other minority subgroups within each population. Similarly, we did not differentiate between race and ethnicity in this study. Various interpretations may exist for the delays seen with specific CPT codes, and individual procedures may be more subject to confounding variables than others. However, these limitations do not detract from the aggregate large-scale data demonstrating delays to surgical treatment of minority patients who require emergent general or vascular surgery but not orthopaedic surgical procedures.

These results suggest that implicit bias may continue to play a role in early and emergent surgical care in the United States, in particular among certain surgical subspecialties, but to a lesser extent within others. Future investigation may help elucidate the factors that contribute to these observed discrepancies. Additional research should focus on systemic influences and challenges with the ultimate goal of expanding access and equalizing care after trauma.

## Supplementary Material
