# Supplementary material for: Bias in Care: Impact of Ethnicity on Time to Emergent Surgery Varies Between Subspecialties
Source: J Am Acad Orthop Surg Glob Res Rev. 2023 Jun 13;7(6):e23.00060. doi: 10.5435/JAAOSGlobal-D-23-00060 (PMC10266510; doi:10.5435/JAAOSGlobal-D-23-00060)
Supplement: Supplementary file 1 [file jagrr-7-e23.00060-s001.docx]

eTable 1: ANOVA results comparing mean time to OR between White and minority populations for top three surgical specialties in NSQIP, measured in days, including individual results by CPT code. Asterisk (*) indicates significant difference (threshold p<0.005) detected relative to White group on post-hoc testing.

| **Top 10 CPT Codes** | **Procedure** | **N** | **White** | | **Black or African American** | | **Asian** | | **American Indian or Alaska Native** | | **Native Hawaiian or Pacific Islander** | | **P** |
| --- | --- | --- | --- | --- | --- | --- | --- | --- | --- | --- | --- | --- | --- |
|  |  |  | Mean | (SD) | Mean | (SD) | Mean | (SD) | Mean | (SD) | Mean | (SD) |  |
| ***General Surgery*** |  | 208125 | **1.03** | (6.88) | **1.36*** | (5.76) | 0.93 | (5.57) | 0.78 | (2.81) | 0.93 | (2.92) | **<0.001** |
| 44970 | Laparoscopic appendectomy | 82415 | 0.36 | (6.82) | 0.35 | (4.65) | 0.41 | (6.55) | 0.16 | (0.65) | 0.25 | (0.84) | 0.868 |
| 47562 | Laparoscopic cholecystectomy | 13001 | 1.26 | (9.25) | 1.11 | (1.7) | 0.99 | (1.38) | 1.05 | (1.7) | 0.87 | (1.52) | 0.868 |
| 44120 | Enterectomy | 9825 | 1.53 | (6.81) | 1.92 | (5.3) | 1.41 | (2.98) | 1.29 | (2.49) | 1.83 | (2.45) | 0.275 |
| 44143 | Partial colectomy with end colostomy | 7345 | 1.74 | (7.41) | 1.97 | (3.69) | 2.13 | (4.68) | 1.75 | (3.03) | 2.9 | (4.86) | 0.859 |
| 44950 | Appendectomy | 6303 | 0.35 | (5.09) | 0.73 | (7.31) | 0.27 | (1.64) | 0.17 | (0.38) | 0.17 | (0.39) | 0.463 |
| **44140** | **Partial colectomy with anastomosis** | 6058 | **1.63** | (4.63) | **2.56*** | (5.47) | 2.01 | (4.05) | 2.26 | (4.79) | 3.06 | (3.84) | **<0.001** |
| 44005 | Incision procedures on the intestines | 4949 | 1.71 | (5.38) | 1.9 | (3.21) | 1.67 | (2.77) | 1.09 | (1.99) | 0.8 | (1.23) | 0.716 |
| 49000 | Exploratory laparotomy | 4641 | 2.36 | (8.42) | 2.58 | (7.59) | 2.44 | (5) | 2.5 | (10.92) | 4.61 | (11.44) | 0.781 |
| 44160 | Partial colectomy with ileocolostomy | 4443 | 1.71 | (7.1) | 1.98 | (3.35) | 1.41 | (2.75) | 1.29 | (2.37) | 0.5 | (0.55) | 0.839 |
| **47563** | **Cholecystectomy with colangiography** | 4350 | **1.06** | (1.47) | 1.27 | (1.85) | 1.27 | (1.51) | **0.59*** | (0.92) | 0.87 | (1.28) | **<0.001** |
| *Orthopaedic Surgery* |  | 22649 | 1.03 | (5.06) | 1.44 | (10.11) | 0.92 | (2.46) | 0.84 | (1.34) | 0.97 | (1.85) | 0.095 |
| 27245 | Treatment pertrochanteric hip fracture (intramedullary) | 4642 | 1.02 | (5.93) | 1.09 | (1.31) | 1.17 | (4.28) | 0.54 | (0.58) | 0.57 | (0.53) | 0.986 |
| 27236 | Open treatment proximal femur fracture | 3724 | 1 | (1.18) | 1 | (0.97) | 1.33 | (2.96) | 1.21 | (2.08) | 1.14 | (1.35) | 0.034 |
| 27125 | Hip hemiarthroplasty | 1661 | 1.13 | (1.18) | 1.52 | (2.01) | 0.97 | (0.78) | 1.14 | (1.46) | 0.62 | (1.06) | 0.040 |
| 27244 | Treatment pertrochanteric hip fracture (plate/screws) | 1555 | 0.93 | (1.27) | 0.74 | (0.76) | 0.68 | (0.53) | 0.88 | (0.83) | 0.6 | (0.55) | 0.488 |
| 27130 | Total hip arthroplasty | 674 | 0.98 | (1.15) | 0.87 | (0.99) | 1.17 | (0.92) | 0.5 | (0.58) | 0 | -- | 0.696 |
| 27814 | Ankle fracture fixation | 556 | 1.31 | (16.33) | 0.79 | (1.49) | 0.46 | (0.88) | 0.33 | (0.58) | 1 | -- | >0.999 |
| 27506 | Femoral shaft fracture intramedullary nail | 403 | 1.98 | (19.7) | 0.94 | (1.39) | 0.36 | (0.48) | 1.67 | (1.53) | -- | -- | 0.969 |
| 27759 | Tibial shaft fracture intramedullary nail | 348 | 0.49 | (0.82) | 0.4 | (0.56) | 0.64 | (0.95) | 0.75 | (0.96) | 0 | -- | 0.807 |
| **27822** | **Trimalleolar ankle fracture fixation** | 324 | **0.48** | (0.7) | **2.28*** | (5.64) | 1 | -- | 0 | -- | 1 | -- | **<0.001** |
| 27792 | Distal fibula fracture fixation | 259 | 0.46 | (1.05) | 0.29 | (0.62) | 0.4 | (0.7) | 0.5 | (0.71) | 0 | -- | 0.931 |
| ***Vascular Surgery*** |  | 18522 | **1.36** | (4.88) | **2.32*** | (8.82) | 1.83 | (5.02) | 1.36 | (2.67) | **3.13*** | (10.38) | **<0.001** |
| 34201 | Embolectomy/thrombectomy, upper leg | 2024 | 0.92 | (5.47) | 0.85 | (1.96) | 0.74 | (1.2) | 0.33 | (0.58) | 1.2 | (1.3) | 0.997 |
| 34802 | Endovascular repair infrarenal aortic aneurysm | 758 | 0.26 | (1.27) | 0.81 | (2.64) | 0.2 | (0.42) | 0 | (0) | -- | -- | 0.041 |
| **34101** | **Embolectomy or thrombectomy, arm** | 751 | **0.73** | (2.06) | 1.02 | (2.56) | 0.64 | (1.6) | 0 | (0) | **10*** | -- | **<0.001** |
| 35301 | Thromboendarterectomy, neck | 716 | 1.95 | (2.52) | 2.7 | (2.91) | 2 | (1.87) | 2.4 | (2.3) | 3 | -- | 0.396 |
| 35082 | Aneurysm repair | 707 | 0.24 | (1.51) | 0.38 | (1.52) | 0.42 | (1.44) | 0 | (0) | 0.5 | (0.71) | 0.968 |
| 27590 | Above-knee amputation | 509 | 3.89 | (7.2) | 6.09 | (12.89) | 2.6 | (3.52) | 3 | (0) | 3.33 | (1.53) | 0.157 |
| 34203 | Embolectomy or thrombectomy, lower leg | 446 | 1.24 | (2.98) | 1.08 | (2.26) | 0.7 | (1.25) | 2 | -- | -- | -- | 0.914 |
| **35371** | **Thromboendarterectomy, common femoral** | 439 | **1.18** | (2.27) | 2.06 | (4.22) | 1.81 | (2.76) | 1 | -- | **18*** | -- | **<0.001** |
| 34803 | Endovascular repair infrarenal aortic aneurysm, modular | 424 | 0.49 | (1.54) | 1.26 | (2.83) | 1.29 | (1.9) | 0 | -- | 0 | -- | 0.046 |
| **27880** | **Below-knee amputation** | 411 | **4.47** | (5.26) | 5.13 | (7.34) | 8.33 | (22.16) | 1 | (0.82) | **15.76*** | (30.77) | **0.002** |

eTable 2: ANOVA results comparing mean time to OR between Non-Hispanic and Hispanic populations for top three surgical specialties in NSQIP, measured in days, including individual results by CPT code. Asterisk (*) indicates significant difference (threshold p<0.005) detected relative to White group on post-hoc testing.

| **Top 10 CPT Codes** | **Procedure** | **N** | **Non-Hispanic** | | **Hispanic** | | **P** |
| --- | --- | --- | --- | --- | --- | --- | --- |
|  |  |  | Mean | (SD) | Mean | (SD) |  |
| ***General Surgery*** |  | 208125 | **1.08** | (6.49) | **0.93*** | (7.9) | **0.005** |
| 44970 | Laparoscopic appendectomy | 82415 | 0.35 | (6.58) | 0.4 | (6.69) | 0.499 |
| 47562 | Laparoscopic cholecystectomy | 13001 | 1.17 | (7.48) | 1.42 | (11.15) | 0.268 |
| 44120 | Enterectomy | 9825 | 1.59 | (6.61) | 1.41 | (3.46) | 0.284 |
| 44143 | Partial colectomy with end colostomy | 7345 | 1.78 | (7.21) | 1.56 | (3.36) | 0.277 |
| 44950 | Appendectomy | 6303 | 0.38 | (5.45) | 0.33 | (1.39) | 0.513 |
| 44140 | Partial colectomy with anastomosis | 6058 | 1.79 | (4.84) | 1.48* | (2.63) | 0.044 |
| 44005 | Incision procedures on the intestines | 4949 | 1.75 | (5.05) | 1.47 | (3.31) | 0.189 |
| 49000 | Exploratory laparotomy | 4641 | 2.42 | (8.42) | 2.13 | (3.88) | 0.290 |
| 44160 | Partial colectomy with ileocolostomy | 4443 | 1.74 | (6.69) | 1.62 | (5.02) | 0.758 |
| 47563 | Cholecystectomy with colangiography | 4350 | **1.05** | (1.49) | **1.19*** | (1.56) | 0.022 |
| *Orthopaedic Surgery* |  | 22649 | 1.05 | (5.56) | 1.08 | (1.71) | 0.645 |
| 27245 | Treatment pertrochanteric hip fracture (intramedullary) | 4642 | 1.02 | (5.91) | 1.12 | (1.33) | 0.428 |
| 27236 | Open treatment proximal femur fracture | 3724 | 1.01 | (1.3) | 1.19 | (1.29) | 0.110 |
| 27125 | Hip hemiarthroplasty | 1661 | 1.13 | (1.2) | 1.31 | (1.54) | 0.381 |
| 27244 | Treatment pertrochanteric hip fracture (plate/screws) | 1555 | 0.91 | (1.24) | 1.02 | (1.09) | 0.485 |
| 27130 | Total hip arthroplasty | 674 | 0.96 | (1.12) | 1.15 | (1.35) | 0.484 |
| 27814 | Ankle fracture fixation | 556 | 1.31 | (16.06) | 0.45 | (0.72) | 0.228 |
| 27506 | Femoral shaft fracture intramedullary nail | 403 | 1.88 | (18.75) | 0.45 | (0.78) | 0.141 |
| 27759 | Tibial shaft fracture intramedullary nail | 348 | 0.51 | (0.83) | 0.25* | (0.44) | 0.006 |
| 27822 | Trimalleolar ankle fracture fixation | 324 | 0.56 | (1.56) | 0.83 | (0.94) | 0.227 |
| 27792 | Distal fibula fracture fixation | 259 | 0.43 | (0.99) | 0.57 | (1.12) | 0.590 |
| ***Vascular Surgery*** |  | 18522 | **1.49** | (5.73) | **2.29*** | (4.29) | **<0.001** |
| 34201 | Embolectomy/thrombectomy, upper leg | 2024 | 0.89 | (5.11) | 1.44 | (4.57) | 0.348 |
| 34802 | Endovascular repair infrarenal aortic aneurysm | 758 | 0.3 | (1.43) | 0.25 | (0.45) | 0.673 |
| 34101 | Embolectomy or thrombectomy, arm | 751 | 0.76 | (2.14) | 0.78 | (1.38) | 0.942 |
| 35301 | Thromboendarterectomy, neck | 716 | 2 | (2.55) | 2.23 | (1.93) | 0.599 |
| 35082 | Aneurysm repair | 707 | 0.23 | (1.46) | 0.96 | (2.44) | 0.168 |
| 27590 | Above-knee amputation | 509 | 4.51 | (9.32) | 3.87 | (5.43) | 0.553 |
| 34203 | Embolectomy or thrombectomy, lower leg | 446 | 1.21 | (2.91) | 1.33 | (2.2) | 0.814 |
| 35371 | Thromboendarterectomy, common femoral | 439 | 1.32 | (2.69) | 1.7 | (2.98) | 0.702 |
| 34803 | Endovascular repair infrarenal aortic aneurysm, modular | 424 | 0.56 | (1.67) | 1.62 | (3.29) | 0.394 |
| 27880 | Below-knee amputation | 411 | 4.84 | (8.07) | 6.03 | (5.91) | 0.308 |
